# A rare case of head (scalp) trauma with foreign body

**DOI:** 10.4103/0019-5545.37677

**Published:** 2007

**Authors:** Gurvinder Pal Singh

**Affiliations:** Department of Psychiatry, Government Medical College and Hospital, Chandigarh, India. E-mail: gpsluthra@rediffmail.com

Sir,

Minor roadside accidents and scalp injuries are very common in developing countries. Treatment of this condition requires assistance from many different disciplines. In the literature, cases of longstanding foreign bodies in the cranium are occasionally reported.[1] The importance of psychiatric evaluation is emphasized in a long-term study of patients with head injuries.[2] Herein, I report a case of foreign body in the scalp and its association with a organic personality disorder.

An 18-year-old unmarried male had a road accident while riding his motorbike. The patient had lacerated injuries on the right parietal region of the head. However, he remained conscious, had no focal neurological signs and his skull X-ray as well as computerized tomographic scan of the brain was unremarkable. A surgical specialist repaired the lacerated wound. Nine months after the incident, his parents sought psychiatric consultation for emerging behavioural problems. Local examination of the scalp revealed chronic discharge of pus from the infected wound (a sinus tract) on the right parietal region. X-ray of the scalp showed a radiolucent area surrounded by dense area on the parietal region of skull. Computed tomography of the brain revealed a calcified lesion in the same region of skull. Surgical debridgement under general anaesthesia and removal of cotton gauze (foreign body) was performed by a neurosurgeon. On detailed psychiatric evaluation, the patient demonstrated a personality change, which was marked by irritability, sudden outburst of anger, labile moods, childishness, voracious eating, irresponsibility and lack of motivation. He had depressive thoughts. At times, his parents had observed that he stopped relating to outsiders as well as family members and remained preoccupied in his own world. As per ICD-10, the patient fulfilled the diagnostic criteria of organic personality disorder.[3] Pharmacological management was done with carbamazepine 200 mg per day. The patient came for regular follow-up and his compliance with treatment was good. The dose of the drug was increased to maximum of 600 mg/day. Family therapy sessions were also planned. Within the first week, the patient reported improvement in his symptomatology. His sleep improved, and severity, frequency and duration of aggressive outbursts diminished. In the next three weeks, his other behavioural problems also improved. He has been reported to be asymptomatic since then for the last 6 months.

In this case report, it seems plausible that the head trauma with a foreign body in the scalp along with subsequent infections may have contributed to the development of organic personality disorder. This is the first case report of a foreign body (cotton gauge) retained for several months in the scalp and its association with such a personality disorder.

There are some interesting things in this case report. The problems started after the head injury. The patient did not have any episode of unconsciousness, convulsion or post-traumatic amnesia. There was no history of drug abuse, chronic physical illness or psychiatric illness prior to the development of organic personality disorder. Minor road accidents and scalp injuries are very common in the developing countries. However, its psychiatric evaluation is often ignored. Behavioural problems, which are linked to the head trauma, and associated conditions are manageable with psychological interventions. Many different treatments have been proposed to improve the conditions that developed in this patient. Carbamazepine is reported to be quite effective in this disorder.[4] Thus, this report suggests that although the head trauma is mild without definite organic signs in the brain, it has a possibility of causing personality change, which may be treatable.

## References

[1] Patel PM, Blackburn TP, Tait MJ, Strong AJ (2004). Cranial blade: Retained for three years. Br J Neurosurg.

[2] Koponen S, Taiminen T, Portin R, Himanen L, Isoniemi H, Heinonen H (2002). Axis I and II psychiatric disorders after traumatic brain injury: A 30-year follow-up study. Am J Psychiatry.

[3] (1992). The ICD-10 Classification of Mental and Behavioural Disorders: Clinical descriptions and diagnostic guidelines.

[4] Lewin J, Summers D (1992). Successful treatment of episodic dyscontrol with carbamazepine. Br J Psychiatry.

